# Pseudoptosis Correction With the 270° Pedicle Reduction Mammoplasty: An Anatomic and Clinical Study

**Published:** 2016-04-28

**Authors:** Matthew R. Zeiderman, Steven Schulz, Charles A. Riccio, Jonathan Nguyen, Saeed Chowdhry, Bradon J. Wilhelmi

**Affiliations:** ^a^University of Louisville School of Medicine, Louisville, Ky; ^b^Division of Plastic and Reconstructive Surgery, Hiram C. Polk Jr. M.D. Department of Surgery, University of Louisville School of Medicine, Louisville, Ky; ^c^Chicago Medical School, Rosalind Franklin University of Medicine and Science, Chicago, Ill

**Keywords:** reduction mammoplasty, breast nipple innervation, breast reduction, 270° pedicle

## Abstract

**Background**: Reduction mammoplasty techniques have evolved considerably. Today, aesthetically pleasing results and preservation of nipple sensation and vascularity are emphasized. Achieving the aforementioned goals for the patient with pseudoptosis remains challenging. **Objective:** We present 270° pedicle reduction mammoplasty as a safe and direct technique for treatment of pseudoptosis to reduce size and improve breast shape. **Methods:** Circumareolar subcutaneous dissection of 10 breasts (5 cadavers) was performed to identify the nerves from the chest wall to the nipple. The trajectory of the nerves to the nipple was identified and dissected to their origin of penetration of the chest fascia. This information provides the basis for lateral chest wall tissue preservation for preserved nipple-areolar innervation, which is incorporated into this technique. Retrospective review of a single surgeon's experience with the 270° pedicle technique for reduction mammoplasty over a 1-year period was performed. **Results:** Anatomic dissection identified 3 to 5 branches of the fourth intercostal nerve to primarily innervate the nipple on 8 of 10 breast dissections. Accessory innervation from the fifth intercostal nerve provided lateral branches to the nipple in 5 of 10 specimens. Five patients underwent reduction mammoplasty with the 270° pedicle technique. No complications were identified. Excellent aesthetic outcomes were achieved on the basis of patient-reported satisfaction and the surgeon's judgment. All patients demonstrated normal nipple sensation at postoperative follow-up. Follow-up at 1 year did not demonstrate recurrence of ptosis/pseudoptosis or change in nipple position. **Conclusions:** The 270° technique for pedicle reduction mammoplasty yields aesthetically pleasing results and symptomatic relief from macromastia and preserves nipple sensation.

Since its inception, reduction mammoplasty techniques have evolved considerably. This is due to evolution in clinical practice and research, which have focused on developing techniques to preserve nipple, skin, and breast parenchyma viability. Previously, surgeons presumed women with macromastia were primarily concerned with breast size and shape over mammary sensation. According to this thought process, the improved aesthetic outcome resulted in an enhanced body image and helped patients feel more sensual. Attempts to accomplish these results lead to the description of many effective methods of reduction mammoplasty, which rely on 3 principles: (1) removal of excess breast tissue; (2) resection of redundant skin to accommodate reduced breast volume; and (3) repositioning of the nipple-areola complex (NAC). One exception to the last principle is in cases of pseudoptosis, or “bottomed-out” breasts, where there is excess gland in the inferior pole of the breast and the NAC remains at or just inferior to the ideal final location at the inframammary crease. In this circumstance, repositioning of the NAC not only is undesirable but also makes an aesthetically pleasing result difficult to attain. Herein the authors describe the 270° pedicle technique for reduction mammoplasty that is ideal for correction of pseudoptosis. This technique is fast and easy to perform, does not require whole breast dissection or transposition of the NAC, maintains a robust pedicle and innervation to the NAC, and produces an aesthetically pleasing result. Innervation of the breast and NAC requires the surgeon to have knowledge of the anatomy and the ultimate goal of preserving the intercostal nerves associated with innervation. This can be achieved in other reduction techniques, but limited breast dissection and tissue disruption with a desired result should be in the forefront of many facets of plastic surgery. The 270° pedicle technique accomplishes both of these goals. We attempted to expand the knowledge of breast and NAC innervation through cadaveric dissections and thus improve aspects of safe breast surgery by presenting this safe and easily reproducible technique.

## METHODS

Dissection of 10 breasts on 5 cadavers was performed at the University of Louisville Fresh Tissue Lab. Circumareolar subcutaneous dissection was performed to identify the nerves from the chest wall to the nipple using 2.5× loupe magnification. Once the trajectory of the nerves to the nipple was identified, the nerves were dissected back to their origin of penetration of the chest fascia.

A retrospective review of a single surgeon's experience at our institution over a 1-year period was also performed (2014-2015). Five patients undergoing mammoplasty with pseudoptosis received the 270° pedicle technique. All patients were nonsmoking females.

### Operative technique

**Figure 1:**
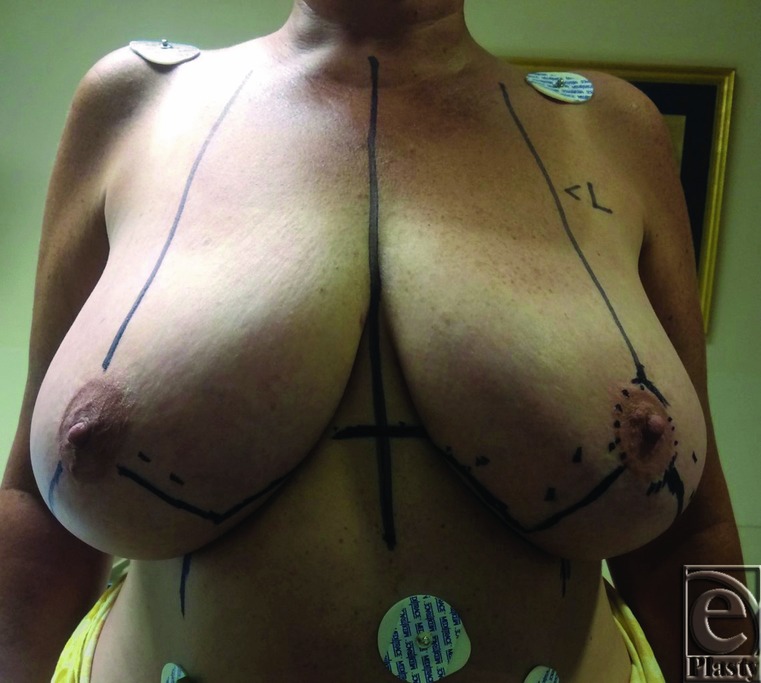
Preoperative photograph of a 50-year-old patient. Markings are made with the patient standing.

**Figure 2:**
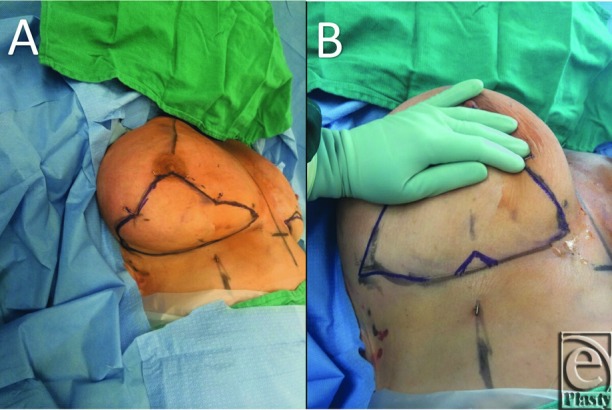
(a) Horizontal limbs of the resection meet the vertical limbs and inframammary fold incision demarcating the mass of tissue to be resected en bloc. (b) Inferiorly based V-flap is placed at the T-conversion of the Wise pattern closure.

**Figure 3:**
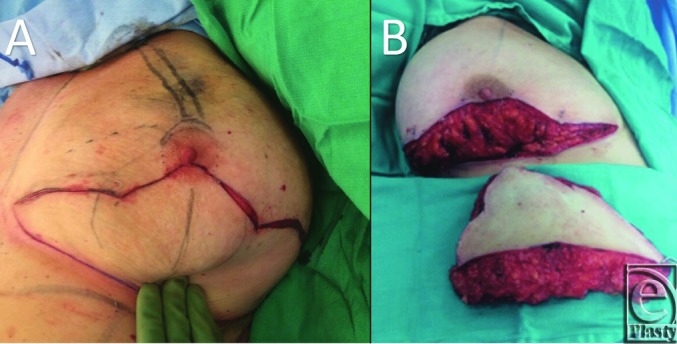
(a) Incision along preoperative markings. (b) Resected breast tissue and the remaining 270° pedicle.

**Figure 4:**
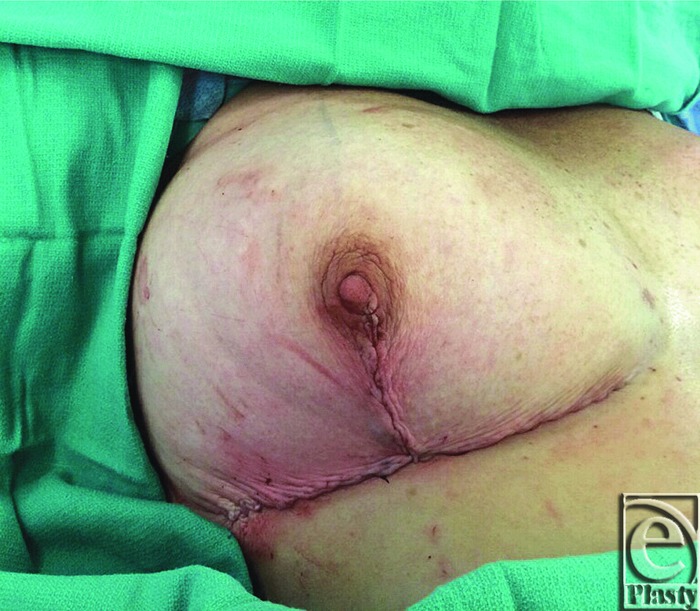
Final approximation of tissue and closure.

Preoperative markings are made with the patient standing. The breast meridian, inframammary fold (IMF), and transposition of the IMF onto the anterior breast skin are marked bilaterally (Fig 1). A 90° angle for the limbs of resection is centered on the breast meridian, with the fulcrum of the angle based on the inferior aspect of the nipple. After confirming that the medial and lateral breast walls will close upon removal of a 90° wedge of tissue, each vertical limb is extended 8 to 10 cm inferolaterally and inferomedially at a 45° angle from the breast meridian, thereby forming a 90° wedge. The horizontal limbs of the resection are extended from the IMF to meet the vertical limbs of resection at about a 120° angle (Fig 2*a*). An inferiorly based dermal inverted V-flap is placed at the intersection of the Wise pattern closure to facilitate wound healing and decrease dehiscence and dog ear deformity (Fig 2*b*). Prophylactic antibiotics are given. It is the surgeon's preference to use cefazolin. The operation is performed under general anesthesia. The patient is prepared and draped in sterile fashion, with the arms abducted and supinated. The marks are scored, and the 270° pedicle is preserved to the NAC (Fig 3*a*). The pedicle is preserved and the inferior breast tissue is removed, preserving the neurovascular supply in the inferolateral and medial breast tissue and along the chest wall (Fig 3*b*). The breast is coned and the wound is closed in a Wise configuration fashion with utilization of the previously described inverted V-flap (Fig 4). The V-flap is de-epithelialized to sit behind the T-point should the T-point have wound-healing complications. If elevation of the NAC is necessary for surgery, a 1- to 2-cm crescent-shaped area can be de-epithelialized to elevate the NAC. This is commonly performed on one side for nipple symmetry.

## RESULTS

The anatomic study results identified 3 to 5 branches of the fourth intercostal nerve to primarily innervate the nipple on 8 of 10 breast dissections. One breast received innervation from the third intercostal nerve and one from the fifth intercostal nerve. Accessory innervation from the fifth intercostal nerve provided lateral branches to the nipple in half of the specimens (Table 1). On the left side, the nerve travels toward the nipple at the 4 o'clock position, whereas it enters at the 8 o'clock position on the right side. The nerve pierces the chest fascia above the fifth rib 3 cm lateral to the border of the pectoralis major muscle and travels through the gland in the prepectoral tissue from inferolateral position toward the nipple.

**Figure 5:**
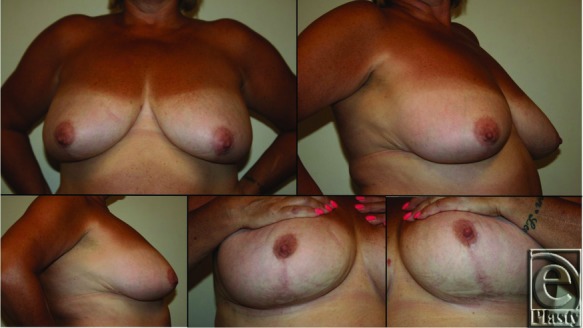
Fifty-year-old woman from Figure 1 at 1-year postoperative follow-up.

Five patients underwent reduction mammoplasty with the 270° pedicle technique. Patients’ age ranged from 32 to 54 years (average 44 years). Individual breast specimen resection weight ranged from 325 to 680 g (average 505 g). There were no short- or long-term complications identified in this case series. Excellent aesthetic outcomes were achieved on the basis of self-reported patient satisfaction at follow-up visits and the surgeon's judgment. There were not any occurrences of delayed wound healing, dehiscence, or infection observed. No necrosis of the NAC resulted. All patients subjectively reported normal nipple sensation at 3-month and 1-year postoperative follow-up. Follow-up at 1 year did not demonstrate any significant recurrence of ptosis/pseudoptosis or change in NAC position as demonstrated by comparison with postoperative photographs (Fig 5).

## DISCUSSION

Breast reduction surgery has evolved considerably through the centuries. Prior to the late 1800s, breast amputation was the procedure performed to eliminate excessively large breasts. Theodore Galliard-Thomas was the first to advocate preservation of some part of the glandular tissue in the 1880s.^1^ The mid-1920s brought the techniques of Lexar and Kraske to transpose the nipple after creating subcutaneous flaps.^1^ Thorek^2^ was the first to perform a free nipple graft for excessive macromastia. In the 1960s, Schwarzman et al^3^ developed the concept of de-epithelialization to maintain the nipple complex on a dermal plexus. Wise built upon Biesenberger's procedure of separating the skin from the gland and transposing the nipple by developing resection patterns to aid in safer, more reliable reductions.^4^^,^^5^ McKissock^6^ later popularized the vertical bipedicle dermal reduction. Inferior pedicle techniques were developed by Robbins,^7^ Courtiss and Goldwyn.^8^ Courtiss^9^ later described the use of liposuction alone as a reduction method. Arie,^10^ Lassus,^11^^-^13 Lejour et al,^14^ and Hall-Findlay^15^ later popularized the vertical reduction.^16^ Primary goals of these procedures through the years have been tissue viability, shape, contour, and scar aesthetics.

An aesthetically pleasing breast embraces the core qualities of minimal scarring, upper-pole fullness, and minimal ptosis.^17^ However, one of the most challenging aspects of breast surgery is consistently providing the desired upper-pole fullness and preventing recurrent ptosis.^18^^-^20

The most popular reduction mammoplasty technique over the past 4 decades has been the inverted T-inferior pedicle technique. Its popularity stems from its easiness to learn and versatility in virtually any situation. However, notable disadvantages include extensive scarring, loss of upper-pole fullness, and the final result having a distorted, “boxy” shape.^17^^,^^21^ While suitable for many breast reductions, it is not ideal for the patient with pseudoptosis who requires resection of breast tissue from the inferior pole of the breast.

In response, the vertical mammoplasty with superior pedicle technique was created to overcome these shortcomings. This short-scar technique reduces both operating times and scar formation, with an aesthetically pleasing final result. However, when used in larger reductions, this technique is often complicated by wound-healing difficulties, unsatisfactory scar formation, and shape distortion such as “bottoming-out” that requires subsequent revision. Hence, this technique is typically limited to reductions of 500 g or less. To overcome the drawbacks of the vertical mammoplasty, the short-scar periareolar inferior pedicle reduction (SPAIR) technique was created.^21^^,^^22^ This technique had many advantages, including the ability to accommodate breast reductions of up to 1500 g and more while also maintaining the pleasing aesthetics of short-scar techniques. The downside of this procedure is that it is technically demanding, requiring accurate flap dissection, and circumvertical skin envelopment around the NAC.^21^ The SPAIR and other periareolar dissection techniques may also result in areolar distortion and widening secondary to tension on the wound.^21^^-^24

Traditional reduction mammoplasty techniques involve nipple transposition to achieve an aesthetically pleasing outcome. However, the circular scarring from periareolar defects decreases the aesthetic quality of the procedure and predisposes to areolar distortion and widening secondary to tension placed on the wound.^21^^,^^23^^,^^24^ Pseudoptosis is characterized by excess breast tissue in the inferior pole of the breast below the NAC. In patient populations whose nipples are already located at the level of the IMF and do not require elevation, the 270° technique is ideal and offers many advantages. Our method of direct excision immediately corrects the characteristic of pseudoptosis of the excess inferior pole. Because of its simplicity, the procedure is easy to learn and fast to perform. It does not require extensive parenchymal dissection, elevation of large breast flaps, or extensive de-epithelialization, which adds to the complexity of the previously described techniques. On the basis of our results, the 270° technique yields aesthetically similar results to the inverted T-technique for the correction of pseudoptosis. However, if nipple elevation is not needed, the periareolar scar is avoided. In addition, this technique can accommodate larger sizes than short-scar vertical mammoplasty techniques, achieving reductions up to 680 g in this study.

The small triangular wedge of de-epithelialized skin left at the center of the inframammary crease incision bolsters vascularity of the incision margins and prevents deep wounds at the T-closure.^12^^,^^25^^,^^26^ If the T-point dehisces, there is dermis underneath that provides a bed for rapid re-epithelialization. This is the most common area of wound breakdown in Wise pattern skin excisions. This V-flap technique is the preference of the surgeon to help alleviate this complication.

Maintenance of a robust pedicle to maintain the NAC is essential. The 270° pedicle technique maintains a vigorous dermoparenchymal pedicle to the NAC from the internal mammary artery and its perforators. This pedicle is unique from other superior pedicle techniques in that the blood supply is provided by superior, medial, and lateral pedicles. This helps avoid dreaded complications of fat and NAC necrosis. Occasionally, traditional pedicles are too long and thus compromise the blood supply to the NAC. When this occurs, patients often require free nipple grafting or the result will be nipple necrosis. Free nipple grafting may subsequently be complicated by NAC necrosis and is frequently complicated by depigmentation.^27^ The 270° pedicle technique for reduction mammoplasty involves minimal or no transposition of the NAC. This prevents NAC necrosis and circular scarring around the NAC frequently encountered with nipple transposition. If elevation of the NAC is necessary in the range of 1 to 2 cm to achieve symmetry, a de-epithelialized crescent-shaped area superior to the lower NAC can be created and approximated to achieve symmetrical nipple position.

The 270° pedicle method avoids full breast dissection. Breast tissue is directly excised only from the inferior quadrant, thereby preventing disruption of blood supply, fat necrosis, and innervation to the NAC. The dissection is beveled laterally by tangentially undermining the pedicle. By preserving 1 to 2 cm of parenchyma and adipose tissue over the muscular fascia of the chest wall and serratus anterior intercostal nerves, the NAC are preserved, thereby keeping nipple sensation intact. This corresponds to the findings of the depth and course of the intercostal nerves from the cadaver dissections done in contribution to this study and in our previous studies of nipple innervation.^28^ Excision of only inferior pole breast tissue maintains the superior pole thickness lost in inferior pedicle techniques. Coning of the breast when approximating the medial and lateral breast tissue creates aesthetically pleasing projection and bolsters superior pole fullness. Finally, we have noticed that direct excision of tissue and avoiding full breast dissection decrease operative time while yielding reproducible results.

The 270° pedicle technique shares some similarities with the recently published superior pedicle technique by Nadeau et al,^29^ but there are several important differences. First, we do not elevate lateral and medial breast flaps, thereby creating a true superior pedicle. Instead, our method maintains a constant 270° pedicle, thereby preserving greater vascularity. Furthermore, our method does not de-epithelialize around the NAC and reposition it upon flap closure. Instead, we rely on only minor positional changes facilitated by a de-epithelialized crescent-shaped area of skin and local rearrangement. As the 270° pedicle technique is indicated for patients with pseudoptosis, where the NAC lies at about the IMF, further repositioning is not indicated, as the IMF is the landmark for NAC positioning in most breast reduction methods.

In general, patients undergoing breast reduction surgery demonstrate high satisfaction due to the improvement in neck, shoulder, and back pain. In addition, the literature now reveals that an evolution has occurred in thinking with regard to what constitutes an ideal outcome. Many advocate that nipple sensation, and not simply aesthetics, is paramount to patient satisfaction. As the nipple is perhaps the most sensitive area of the breast, it serves a significant role in a woman's sexual life. The anatomic studies by our group and others have demonstrated the primary innervation to the NAC to derive from the fourth intercostal nerve in the inferolateral quadrant of the breast.^29^^,^^30^^-^33 By avoiding full breast dissection, this method avoids disruption of the primary as well as accessory innervation to the NAC.

Frequently, the plastic surgeon must individualize therapy to the patient. A fixed procedure does not always apply to every clinical scenario. These patients were selected for this procedure on the basis of the diagnosis of macromastia with pseudoptosis and an NAC location at or around the IMF, therefore requiring only resection of skin and parenchyma from the inferior quadrant of the breast without NAC elevation. Adhering to principles of technique and knowledge of anatomy frequently serves as a foundation for the reconstructive surgeon when planning procedures. This study can aid the novice and experienced surgeon in obtaining quality outcomes in terms of not only aesthetics but also function.

## CONCLUSIONS

Attaining aesthetically pleasing results, providing symptomatic relief from macromastia, and maintaining nipple sensation are valuable goals in breast surgery. The 270° pedicle technique for reduction mammoplasty effectively fulfills these goals for the patient with pseudoptosis. This technique is quick to perform and easy to learn. This study can aid the novice and experienced plastic surgeon in obtaining quality aesthetic and functional outcomes.

## Figures and Tables

**Table 1:** Cadaveric dissection of nipple innervation*

Specimen	Side	ICN	Accessory ICN	No. of branches
1	L	4	3	3
2	L	4	5	3
3	R	4	5	5
4	R	5	5	5
5	L	4	3	4
6	R	4	3	4
7	R	3	4	4
8	L	4	5	3
9	L	4	5	5
10	R	4	3	3

*Specimen data for 10 dissections equally distributed between left and right sides. Eight of 10 showed primary innervation from the fourth ICN. Accessory innervation came from ICN 3 to 5. The primary nerve supplied 3 to 5 branches to the nipple. ICN indicates intercostal nerve; L, left; R, right.
